# The effect of subcutaneous and sublingual birch pollen immunotherapy on birch pollen–related food allergy: a systematic review

**DOI:** 10.3389/falgy.2024.1360073

**Published:** 2024-06-06

**Authors:** E. J. J. Kallen, P. M. J. Welsing, J. M. Löwik, R. Van Ree, A. C. Knulst, T. M. Le

**Affiliations:** ^1^ Department of Dermatology/Allergology, University Medical Center Utrecht, Utrecht University, Utrecht, Netherlands; ^2^ Departments of Experimental Immunology and Otorhinolaryngology, Amsterdam University Medical Centers, Amsterdam, Netherlands; ^3^ Centre of Translational Immunology, University Medical Center Utrecht, Utrecht University, Utrecht, Netherlands

**Keywords:** food allergy, pollen food allergy syndrome (PFAS), BPFA, birch pollen allergy, sublingual immunotherapy (SLIT), subcutaneous immunotherapy (SCIT), allergen immune therapy (AIT), systematic review

## Abstract

**Background:**

Birch pollen–related food allergy (BPFA) is the most common type of food allergy in birch-endemic areas such as Western and Central Europe. Currently, there is no treatment available for BPFA. Due to the cross-reactivity between birch pollen and a range of implicated plant foods, birch pollen allergen immunotherapy (AIT) may be effective in the treatment of BPFA. In this study, we systematically evaluate the effectiveness of birch pollen–specific subcutaneous or sublingual immunotherapy in treating BPFA.

**Methods:**

A search was performed in the PubMed, Embase, and Cochrane libraries. Studies were independently screened by two reviewers against predefined eligibility criteria. The outcomes of interest were changes in (1) severity of symptoms during food challenge, (2) eliciting dose (ED), and (3) food allergy quality of life (FA-QoL). The validity of the selected articles was assessed using the revised Cochrane risk of bias tool. We focused on studies with the lowest risk of bias and considered studies with a high risk of bias as supportive. Data were descriptively summarized.

**Results:**

Ten studies were selected that included 475 patients in total. Seven studies were categorized into “high risk of bias” and three into “moderate risk of bias.” The three moderate risk of bias studies, with a total of 98 patients, reported on severity of symptoms during challenge and on the ED. All three studies had a control group. Compared to the control group, improvement in severity of symptoms was observed during challenge in two out of the three studies and on the eliciting dose in one out of three. Only one study investigated the effect of birch pollen AIT on FA-QoL, showing that there was no significant difference between patients receiving subcutaneous immunotherapy or a placebo. Of the seven supportive studies, four had a control group and of those, three showed improvement on both severity of symptoms and ED. None of the supportive studies investigated the effect of the therapy on FA-QoL.

**Conclusion:**

This systematic review shows that there is not enough evidence to draw firm conclusions about the effect of AIT on BPFA. Future research is warranted that uses robust clinical studies that include long-term effects, QoL, and multiple BPFA-related foods.

## Introduction

In Europe, the rate of prevalence of birch pollen sensitization ranges from approximately 8% to 16%, and climate change is likely to cause this number to increase over time (1, 2). Pollen derived from the *Betulaceae* and *Fagaceae* family constitutes the most prominent source of tree pollen in Western and Central Europe (2). The major birch pollen allergen, Bet v 1, is a PR-10 protein whose homologous structures are present in a large number of plant foods (3). Due to cross-reactivity between Bet v 1 and these homologs in foods, approximately 70% of birch pollen–allergic patients report allergic reactions to foods, commonly referred to as birch pollen–related food allergy (BPFA) (3). BPFA is the most common type of food allergy in Western and Central Europe involving many different foods and food groups, for example, *Rosaceae* fruits such as apples and peaches, tree nuts such as hazelnuts and walnuts, and vegetables such as carrots, celeriac, and soy (2, 4).

Symptoms of BPFA are usually mild and restricted to the oral cavity; hence, they are often referred to as oral allergy syndrome (OAS). However, sometimes more severe allergic reactions with cardiovascular symptoms, or even anaphylaxis, can occur involving some foods, for example, soy protein–containing food (2, 5).

To date, no treatment is available for BPFA. The evidence for the effectiveness of oral immunotherapy to foods relating to BPFA is sparse (6). Because birch pollen is the primary sensitizer in BPFA, birch pollen AIT has often been considered possibly effective also in the treatment of BPFA (3). However, there is no evidence supporting this, and it is even hypothesized that due to insufficient homology between Bet v 1 and plant food allergens, it is not possible to alleviate BPFA symptoms (7). Both subcutaneous (SCIT) and sublingual (SLIT) immunotherapy are available for birch pollen allergy, but their effectiveness for treating associated food allergies remains a matter of debate (8, 9).

The aim of this review was to systematically evaluate the effect of birch pollen–specific SCIT and SLIT on BPFA with regard to severity of symptoms during challenge, eliciting dose (ED), and food allergy-related quality of life (FA-QoL).

## Methods

### Eligibility criteria, information sources, and search

A systemic search strategy (Supplementary Material S1) was developed by combining synonyms for the patient population and intervention using both keywords and medical subject headings. The patient population consisted of those with birch pollen allergy *and* birch pollen–related food allergy; subcutaneous *or* sublingual birch pollen–specific immunotherapy was used as an intervention. Our search was performed in the PubMed, Embase, and Cochrane libraries on 3 November 2022.

### Study selection

Citations from the PubMed, Embase, and the Cochrane libraries were imported into the Rayyan tool for removing duplicates and for screening. Two authors (JL and EK) independently screened the titles and abstracts. When a paper was deemed possibly relevant, the full text was also independently screened by these two authors. Selection was based on consensus, and discrepancies were resolved by two other authors (TL and PW). English language articles that met the following criteria were included: (1) subjects with a birch pollen allergy, (2) subjects with BPFA for at least one food, (3) those with either birch pollen–specific SCIT or birch pollen–specific SLIT as an intervention, and (4) studies in which the effectiveness of this treatment was evaluated in terms of food challenge. Studies focusing on food allergy immunotherapy, non-original studies (editorials and expert opinions), conference abstracts, case studies, and animal studies were excluded. Reviews were also excluded, but they were used to obtain additional articles of interest based on reference checking.

### Data extraction

Two authors (JL and EK) independently recorded the characteristics of the selected studies using a predefined checklist, comprising the following items: (1) study information (first author, year of publication, and country in which the study was performed); (2) study design [randomized controlled trial (RCT) or comparative/single-arm prospective cohort]; (3) type of food challenge [double-blind, placebo-controlled food challenge (DBPCFC) or open food challenge (OFC)]; (4) type of food; (5) treatment group characteristics (number of patients and type of immunotherapy), if applicable; (6) control group characteristics (number of patients and type of control); (7) timepoint when the outcome was measured; and (8) type of reported outcomes. The extracted outcome measurements were changes in (1) severity of symptoms during challenge, (2) eliciting dose, and (3) food allergy–related quality of life. Improvement in the eliciting dose was defined as the percentage of patients who could tolerate at least one higher dose without symptoms during the last-performed food challenge compared with baseline.

### Risk of bias assessment

The validity of the selected studies was assessed using the revised Cochrane risk of bias tool (RoB2) (10), which evaluated five domains of bias: D1, the randomization process; D2, deviations from intended interventions; D3, missing outcome data; D4, measurement of the outcome; and D5, selection of the reported result. The following information was assessed: D1, performance of randomization, observed baseline differences in patient characteristics, and concealment of allocation sequence; D2, awareness of the assigned intervention, deviations from the intended intervention due to the trial context, and whether the analysis used to estimate the effect of assignment to the intervention was appropriate; D3, availability of outcome data; D4, whether the method of measuring the outcome was appropriate, comparable between intervention groups, and insensitive to awareness of the received intervention; and D5, whether data analysis was prespecified. Each of the questions in the domains could be answered with “yes,” “probably yes,” “probably no,” “no,” and “no information, which led to a risk of bias per domain classified as ‘low’, ‘some concerns’, or ‘high’”. Furthermore, the overall risk of bias was determined. Single-arm studies scored high for domains 1 and 2 because there was no control group/randomization (treatment effect estimates concerned pre/post-treatment differences) and patients and caregivers were aware of the received intervention.

### Synthesis of results

Due to evident heterogeneity between the studies in terms of design, timepoint when outcome was measured, type of food, type of immunotherapy, type of control group, and availability and measurement of the five outcomes, it was considered inappropriate to pool the results. Therefore, a qualitative synthesis of the available results was performed without producing a formal statistical summary. Studies with the lowest risk of bias with a control group were considered the most important, while studies with the highest risk of bias without a control group were considered only supportive. Furthermore, a distinction was made between the direction of the effect (positive, no effect, and negative) and the size of the effect in case of an effect (large or small). Based on clinical interpretation, the size of the effect was considered large when there was an improvement of at least 20%. In the summary of the effect of birch pollen AIT on BPFA, only objective results are shown when a study reported on both subjective and objective results, because these are more reliable.

## Results

### Study selection

**Figure 1:**
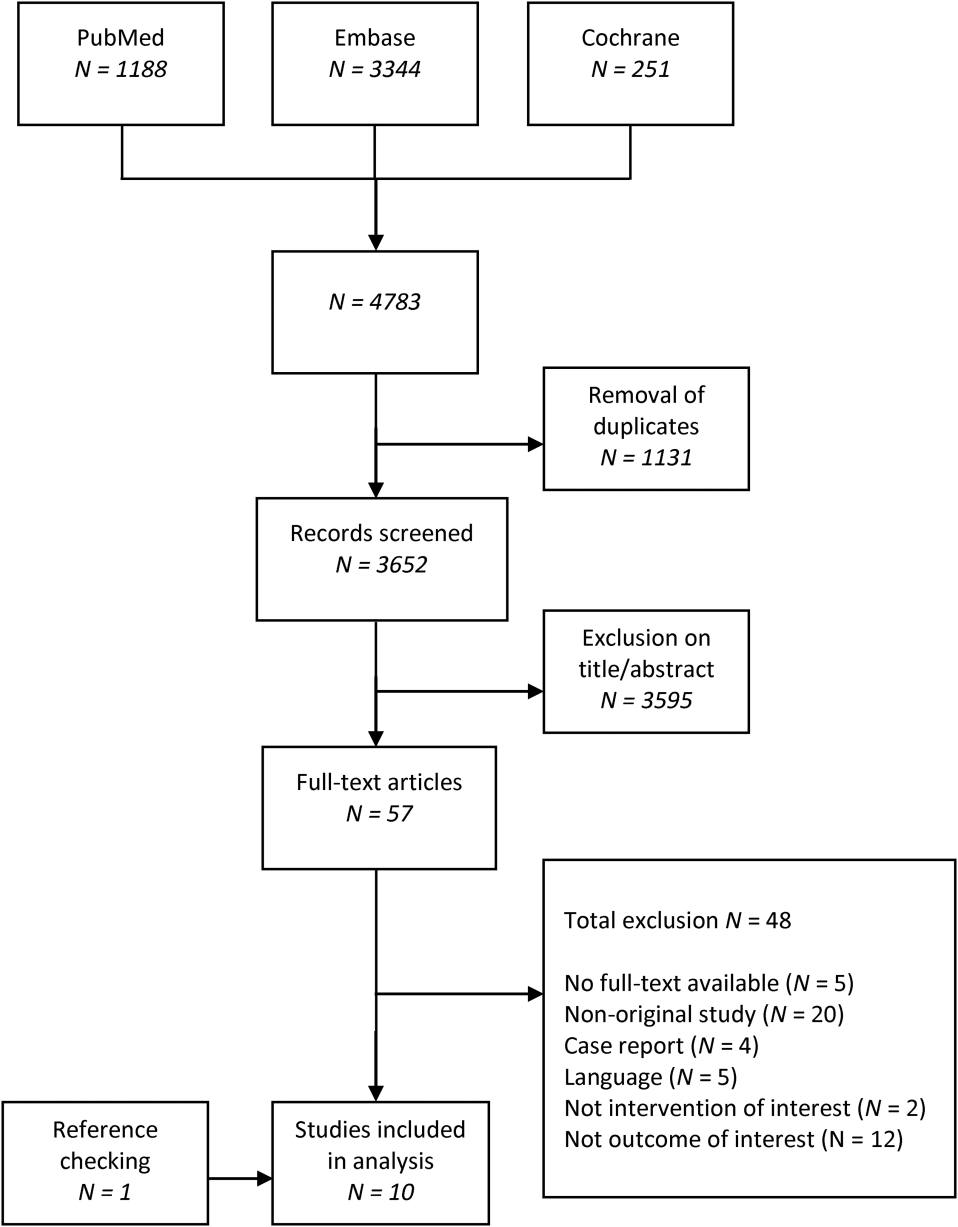
A flowchart of literature search in PubMed, Embase, and Cochrane.

Our search yielded 3,652 unique articles (Figure 1). After screening the articles by title, abstract, full text, and reference checking, 10 articles were included.

### Study characteristics

Details of the 10 selected studies can be found in Table 1. All the studies were conducted in Europe. In total, there were five RCTs (of which one was a sub-study of an RCT) and five prospective cohort studies. A control group was used in all RCTs and in two prospective cohorts. In four out of five RCTs, the control group was a placebo group. In the other studies, the control group consisted of patients without AIT. In the studies without a control group, a pre/post-AIT comparison of the outcome(s) was made.

**Table 1:** Characteristics of the included articles.

A	Study information (first author, year, country)	Study design	Type of challenge	Type of food	Treatment group characteristics	Control group characteristics	Time-measured outcome (months)
1	Treudler, 2017 (11), Germany and Switzerland	RCT	DBPCFC	Soya	*N* = 38 SCIT (Allergopharma) Bet v 1	*N* = 18 Placebo	12
2	Van Hoffen, 2011 (12), The Netherlands	RCT	DBPCFC	Hazelnut	*N* = 10 SCIT (Alutard SQ) Birch pollen extract	*N* = 9 Placebo	12
3	Bolhaar, 2004 (13), The Netherlands	RCT	DBPCFC	Apple (Golden Delicious)	*N* = 13 SCIT (Alutard SQ) Birch pollen extract	*N* = 10 Without AIT	12
4	Hansen, 2004 (14), Denmark	RCT	OFC	Apple (Golden Delicious)	*N* = 28 – SCIT: *N* = 16 (Phostal) – SLIT: *N* = 12 (Staloral) Both Bet v 1	*N* = 14 Placebo	24
5	Till, 2020 (15), United Kingdom	RCT (sub-study)	OFC	Apple (Golden Delicious)	*N* = 61 SLIT (12 SQ-Bet, ALK) Birch pollen extract	*N* = 63 Placebo	6.5–9.5
6	Asero, 1998 (16), Italy	Prospective cohort	OFC	Apple (Golden Delicious)	*N* = 49 SCIT (Allergopharma retard (*N* = 33) or Bayer Alhydrox (*N* = 16)) Both birch pollen extract	*N* = 26 Without AIT	12–48
7	Bucher, 2004 (17), Switzerland	Prospective cohort	OFC	Apple (Golden Delicious) and hazelnut	*N* = 15 SCIT (Alutard SQ) Birch-hazel-alder pollen extract (*N* = 9), ash pollen (*N* = 3), grass/rye/birch pollen extract (*N* = 2), birch pollen extract (*N* = 1)	*N* = 12 Without AIT	12
8	Van der Valk, 2020 (18), The Netherlands	Prospective cohort	OFC	Apple (not specified)	*N* = 5 SCIT (Alutard SQ) Tree mix	—	24
9	Bergmann, 2008 (19), Germany	Prospective cohort	OFC	175 foods that induced BPFA symptoms (mainly apple and hazelnut)	*N* = 102 SLIT [B.U. Pangramin (*N* = 81)] Tree mix	—	12
10	Kinaciyan, 2007 (20), Austria	Prospective cohort	OFC and DBPCFC	Apple (Golden Delicious)	*N* = 20 SLIT (B.U. Pangramin) Bet v 1	—	12

A, article number; *N*, Number.

Altogether, 475 patients were analyzed, of whom 320 received AIT and 152 did not. Of the 320 patients who received AIT, 127 served as their own controls. Six studies focused on SCIT, three on SLIT, and one on either SCIT or SLIT. The last study was a three-arm study, in which patients receiving SCIT or SLIT or a placebo were compared. An OFC was performed in six studies, a DBPCFC in three studies, and both OFC and DBPCFC in one study.

During treatment, outcomes were reported at timepoints between 6.5 and 48 months after the start of treatment, but in 6 out of 10 studies, they were reported at 12 months.

### Risk of bias

The overall risk of bias was moderate in three studies and high in seven. Therefore, we focused on the three studies with the lowest risk of bias; the remaining studies were considered to be supportive. The overall risk of bias was mainly high because of issues with domain 2, “Deviations from the intended interventions,” which were attributed to no correction of prognostic factors in non-randomized studies. Details of the assessment per outcome reported are presented in Table 2.

**Table 2:** Risk of bias assessment^a^.

A	Comparator	Outcome	D1	D2	D3	D4	D5	Overall
1	Placebo	Severity of symptoms during challenge	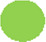	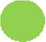	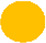	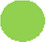	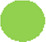	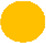
1	Placebo	Eliciting dose	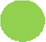	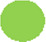	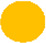	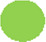	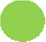	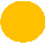
1	Placebo	Food allergy–related quality of life	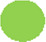	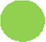	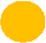	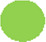	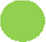	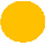
2	Placebo	Severity of symptoms during challenge	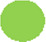	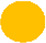	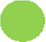	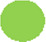	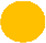	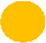
2	Placebo	Eliciting dose	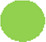	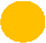	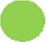	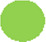	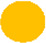	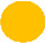
3	Control	Severity of symptoms during challenge	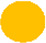	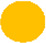	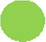	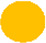	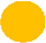	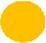
3	Control	Eliciting dose	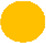	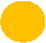	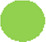	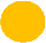	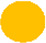	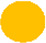
4	Placebo	Severity of symptoms during challenge	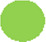	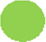	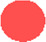	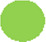	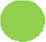	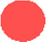
4	Placebo	Eliciting dose	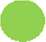	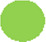	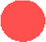	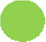	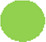	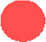
5	Placebo	Severity of symptoms during challenge	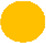	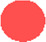	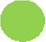	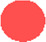	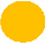	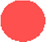
5	Placebo	Eliciting dose	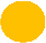	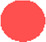	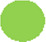	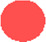	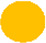	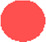
6	Control	Severity of symptoms during challenge	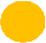	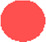	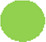	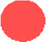	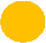	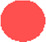
6	Control	Eliciting dose	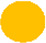	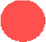	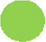	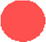	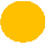	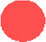
7	Control	Severity of symptoms during challenge	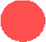	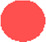	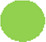	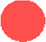	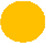	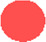
7	Control	Eliciting dose	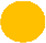	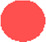	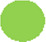	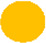	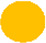	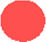
8	Baseline	Eliciting dose	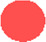	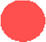	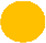	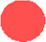	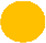	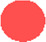
9	Baseline	Severity of symptoms during challenge	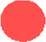	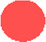	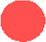	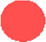	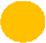	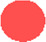
9	Baseline	Eliciting dose	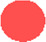	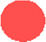	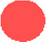	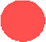	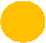	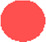
10	Baseline	Severity of symptoms during challenge	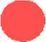	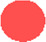	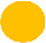	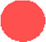	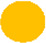	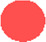

A, article number; D1, randomization process; D2, deviations from the intended interventions; D3, missing outcome data; D4, measurement of the outcome; D5, selection of the reported result. 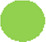, low risk of bias; 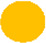, moderate risk of bias; 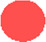, high risk of bias.

^a^ RoB2 was used for the risk of bias assessment (10).

### Study outcomes

A summary of the study results regarding the outcomes of severity of symptoms during challenge and ED is provided in Tables 3 and 4. In both tables, a distinction is made between studies with the lowest and the highest risk of bias and those with or without a control group. In studies with a control group, treatment effects pertained to the comparison between treatment and control groups, and in studies without a control group, treatment effects related to changes from baseline. Henceforth, for the three studies with the lowest risk of bias, only the comparison between the treatment and the control groups is discussed subsequently. Within these studies, a total of 61 patients received AIT and 37 did not.

**Table 3:** Overview of the effect of birch pollen AIT on severity of symptoms during challenge and eliciting dose.

**A**	**Change in severity of symptoms during challenge**	**Change in eliciting dose (g)**
Comparative studies: numbers are treatment vs. control unless mentioned otherwise		
Studies with a moderate risk of bias		
1	– Subjective symptoms from 95% to 67% vs. from 83% to 60% – Objective symptoms from 82% to 24% vs. from 78% to 47% – No significant difference	– Mean ED subjective symptoms from 2.2 to 4.7 vs. from 0.7 to 2.2 – Mean ED objective symptoms from 4.7 to 24.7 vs. from 2.2 to 24.7 – No significant difference
2	– Numbers not mentioned – No significant difference	– Mean ED from 0.65 to 0.65 vs. from 0.10 to 1.00 – No significant difference – Median ED – Higher in 40% vs. 67% – Unchanged in 20% vs. 11% – Lower in 40% vs. 22% – No significant difference, *p*-value not specified
3	– Improvement: 69% vs. 0% – Unchanged: 31% vs. 90% – No performed DBPCFC: 0% vs. 10%	– Increased by factor 24 vs. no change – HD: 23% vs. 0%
Studies with a high risk of bias		
4	SCIT vs. SLIT vs. placebo – Improvement in the mean symptom score (severity 0–3) from 1.8 to 1.2 vs. from 1.5 to 1.2 vs. from 2.0 to 1.5 (only in placebo group significant improvement)	SCIT (*N* = 10) vs. SLIT (*N* = 4) vs. placebo (*N* = 10) – HD: 10% vs. 0% vs. 20%
5	– More patients with a lower VAS score at each dose than in the control group *No OFC at baseline*	– HD: 75% vs. 68% *No OFC at baseline*
6	– Improvement in 84% vs. 0% – *p* < 0.001 *The treatment group used OFC; the control group used a questionnaire*	– HD: 45% vs. 0% *The treatment group used OFC; the control group used the questionnaire*
7	– Subjective symptoms from 60% to 67% vs. from 83% to 92% – Objective symptoms from 20% to 0% vs. from 0% to 42%	– Mean ED for objective signs from 12.6 to 32.6 vs. from 9.8 to 8.5 – HD: 13% vs. 0% – Percentage of patients with changed ED – Higher: 87% vs. 8% – Unchanged: 13% vs. 0% – Lower: 0% vs. 92% – *p* < 0.01
Singe-arm studies: numbers are endpoint vs. baseline unless otherwise mentioned		
Studies with a high risk of bias		
8		– Change in the median ED (steps): from 1.4 to 6.0 – HD: 40% vs. 0% – Percentage of patients with changed ED: – Higher: 80% – Unchanged: 20%
9	– >50% improvement in 77% – *p* < 0.0001 *At baseline questionnaire and at endpoint OFC*	Number of tolerated ingested food types from the standard list: – From 41% at week 4 to 86% at month 12
10	– Change in mean reported VAS (range 0–10) * OFC from 2.8 to 2.9 * DBPCFC from 4.5 to 4.1 – No significant difference	

A, article number; HD, ingestion of highest dose without symptoms during the last-performed challenge; *N*, number.

**Table 4:** Summary of the effect of birch pollen AIT on severity of symptoms during challenge and eliciting dose within the treatment group (change from baseline) and between the treatment and the control group.

Outcome measurement	Severity of symptoms during challenge		Eliciting dose	
Comparison of the treatment group with	Baseline	Control	Baseline	Control
Article number				
Studies with a moderate risk of bias				
1	++^a^	++^a^	++	
2				−
3	++	++	++	++
Studies with a high risk of bias				
4	+			−
5		NM		+
6^b^	++	++		++
7	++^a^	++^a^	++	++
8			++	
9^c^	++		++	
10				

NM, not mentioned. Direction of the effect: 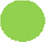, positive effect; 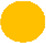, no effect; 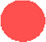, negative effect. The size of the effect in case a positive or negative effect was observed: <20% difference +/−; >20% difference ++/−−; when the size of the effect was not mentioned, the abbreviation NM was used.

^a^ Only the objective symptoms are shown.

^b^ The treatment group used OFC; the control group used a questionnaire.

^c^ At baseline a questionnaire and at endpoint OFC.

Birch pollen AIT seems to have had a positive effect on severity of symptoms during challenge in the three studies with a moderate risk of bias; two showed a positive effect (11, 13) on severity of symptoms and one no effect (12) (Tables 3, 4). The first study distinguished between objective and subjective symptoms and showed a decrease in both objective and subjective symptoms in both the treatment and the control groups. Although there was no significant difference in the decrease, the patients in the treatment group tended to have a higher numerical decrease in objective symptoms than those in the control group (11). The second study with a positive effect showed a rate of reduction in symptoms of 69% in the treatment group vs. 0% in the control group (13).

The study with no effect only mentioned that there was no significant difference in symptoms during challenge; no numbers were mentioned (12).

The effect of birch pollen AIT on the eliciting dose is unclear. Of the three studies with a moderate risk of bias, one showed a positive effect (13), one no effect (11), and one a negative effect (12) on the ED (Tables 3, 4). The study with a positive effect showed a 24-fold increase in the eliciting dose in the treatment group vs. no change in the control group. Furthermore, 23% of the patients in the treatment group vs. 0% of the patients in the control group reached ingestion of the highest dose without symptoms during the last challenge (13). The study with a negative effect showed a baseline mean ED that did not change in the treatment group but increased from 0.10 to 1.00 g in the control group (12).

Further research is needed to assess the effect of birch pollen AIT on FA-QoL. Only one study with a moderate risk of bias investigated the effect of birch pollen AIT on FA-QoL (11). This study used the validated food allergy quality of life questionnaire—adult form (FAQLQ-AF) and showed that there was no significant difference between patients receiving SCIT or a placebo. No studies with a high risk of bias investigated the effect of birch pollen AIT on FA-QoL

Supporting studies showed mostly positive effects. The seven studies with a high risk of bias were considered supportive, of which four (14–17) included a control group and three (18–20) did not (Table 4). When there was a positive effect on severity of symptoms during challenge, there was also a positive effect seen on the ED. Of the studies with a control group, three (15–17) showed a positive effect on severity of symptoms and the ED with mostly a large effect size and one (14) no effect on severity of symptoms during challenge and a negative effect with a small effect size on the eliciting dose. Of the studies without a control group, two (18, 19) showed a positive effect with a large effect size and one (20) no effect.

Overall, these high risk of bias studies supported the moderately positive effect of birch pollen AIT on severity of symptoms during challenge and indicated that the effect on the eliciting dose was more likely to be positive.

## Discussion

Due to the small number of included studies and the moderate to high risk of bias in these studies, this systematic review primarily shows that there is not enough evidence to conclude that AIT reduces BPFA. There may be a positive effect on the severity of symptoms during challenge. The effect on the eliciting dose is, however, unclear, and there is not enough information to draw a conclusion about the effect of birch pollen AIT on FA-QoL.

### The evidence of the effect of birch pollen AIT on BPFA is of low quality

As mentioned previously, 7 (14–20) of the 10 studies had a high risk of bias, mostly due to domain 2, “Deviations from the intended interventions,” followed by domain 4, “Measurement of the outcome.” The high risk of bias in domain 2 was due to studies without a control group and studies with a control group but not adjusted for prognostic factors when no randomization was performed. The high risk of bias in domain 4 was attributed to the fact that, among others, studies performed an OFC only at the end of the study and not at the start (15, 19) or used a method in the treatment group that was different from that in the control group (16). Because most studies had a high risk of bias, the quality of evidence was low. To obtain the best possible estimation of effectiveness, we decided to use the three studies with the lowest risk of bias for the assessment and the studies with a high risk of bias only as supporting evidence.

### More research is needed to investigate the effect of birch pollen AIT on BPFA

Birch pollen AIT seems to have a positive effect on BPFA as evidenced by an alleviation of symptoms during challenge, and it remains unclear whether there is also a positive effect on the ED. That some studies found no or even a negative effect could be attributed to the fact that the included patients might not have had a pure BPFA but also a primary food allergy that did not reduce or even worsen during treatment and/or to the fact that there was an imbalance in the groups in this respect. Nowadays, by measuring both the PR-10 and the non-PR-10 components, it has become possible to differentiate between a pure BPFA and a primary food allergy (21). The study that showed that there was no effect on symptoms during challenge and a negative effect on the eliciting dose measured only Cor a 1 and Cor a 8 but not Cor a 9 and 14 (12). Therefore, it was unclear whether only patients with pure BPFA were included.

Another reason could be that the follow-up period was too short. In general, the effect of immunotherapy increases with a longer duration (22, 23). All three studies with the lowest risk of bias reported their results only after 1 year of AIT use.

### Studies investigating the effect of birch pollen AIT on food allergy–related quality of life are lacking

Patient-reported outcome measures (PROMs) are often the best way of measuring patient symptoms and quality of life and can help reduce observer bias (24). Unfortunately, only one study investigated the effect of birch pollen AIT on the patient-reported outcome “FA-QoL” using the validated FAQLQ-AF questionnaire and showed that there was no significant difference between patients receiving SCIT or placebo at endpoint (11). As the primary burden on patients living with food allergy is a reduced QoL, treatment success in trials should also be defined by an improved QoL (25, 26). Future studies, investigating the effect of birch pollen AIT on BPFA, should therefore include QoL as an outcome.

### It is not possible to evaluate potential differences between birch pollen SCIT and birch pollen SLIT on BPFA

In total, six studies investigated the effect of birch pollen SCIT (11–13, 16–18), three that of birch pollen SLIT (15, 19, 20), and one that of both birch pollen SCIT and SLIT (14) on BPFA. Because of the small number of studies and the high risk of bias, there is too little evidence to draw a conclusion.

### Effect of birch pollen AIT on different foods

In our systematic review, we found that almost all studies investigated the effect of AIT with either hazelnut or apple as a type of food. This is not surprising, as hazelnut and apple allergies are among the top three birch pollen–related food allergies reported in birch pollen–endemic areas (27). However, patients with BPFA are mostly allergic to multiple types of fruits, vegetables, and nuts. The effect on other foods remains unknown. Therefore, it is important to evaluate the effect of AIT on a broader range of BPFA foods.

We expected a better therapeutic effect of birch pollen AIT on foods with PR-10 components that are more homologous to Bet v 1. However, although the PR-10 components of apple and hazelnut are more homologous to Bet v 1 than those of soy (28), this review showed a better effect of birch pollen AIT on soy (11) than on hazelnut allergy (12). To confirm this hypothesis, more studies are needed that compare the effect of birch pollen AIT on multiple foods related to BPFA.

### Further studies are needed to investigate the long-term effects of birch pollen AIT on BPFA

It is unknown how long the effects last after one discontinues AIT. Most of the included studies measured the outcome after 12 months of the start of birch pollen AIT, but none showed results after discontinuation of AIT. In 2003, Asero conducted a prospective cohort study to evaluate the long-term effect of birch pollen–specific SCIT on apple allergy after treatment cessation (29). In this study, 21 BPFA patients who discontinued birch pollen SCIT could tolerate apple. However, the effect appeared to decrease over time, since after 30 months of discontinuation, only 52% of the patients remained symptom-free. Further studies are needed to investigate the long-term effects of birch pollen AIT on BPFA so that clinicians can advise patients appropriately.

### Positive effect of other immunotherapies on plant food allergy

Apart from the effect of birch pollen AIT on BPFA, several studies showed the effect of other immunotherapies on plant food allergy. Two studies reported about an effective treatment of birch pollen–related apple allergy. Kinacyian et al. (30) showed that patients receiving SLIT with rMal d 1 required a significantly higher dose of rMal d 1 to induce OAS compared with the group that received rBet v 1 and placebo (*p* = 0.001), and Kopac et al. (31) showed that apple consumption induced a transient tolerance. Furthermore, studies from Japan (32) and Italy (33) reported the positive effects of Japanese cedar pollen–based SCIT and grass pollen SLIT, respectively, on plant food allergy. All of the above studies showed promising approaches for the effective treatment of plant food allergy, but these results should be confirmed before they are used in clinical practice.

### Strengths and limitations

Due to the high risk of bias and the heterogeneity of the included studies in terms of, among other elements, the study design, type of food, type and dose of immunotherapy, and method of assessing response to treatment, it was not possible to pool the results. Furthermore, symptoms were often not specified, and none of the studies reported the minimal clinically important difference (MCID) to define improvement, which made it difficult to interpret whether the differences found were clinically relevant (34). To provide the most reliable results, we focused on the three studies with the lowest risk of bias. Because of this selection, the total number of patients was small, with the total number of patients receiving AIT being 61 and those not receiving AIT being 37. However, the strengths of this review included its comprehensive search and methodological rigor, which also took into account patient-reported outcomes. This factor made this review the first systematic one to show the effect of BPFA on clinical and patient-reported outcomes.

## Conclusions

To our knowledge, this is the first systematic review that evaluates the effect of birch pollen AIT on BPFA. Due to the low number of studies that fulfilled the inclusion criteria, a moderate to high risk of bias in these studies, and the low number of included patients per study, the level of evidence is low. The three studies with the lowest risk of bias showed that there might be a positive effect on severity of symptoms during challenge, but there was an unclear effect on the eliciting dose, and there was not enough information available to draw a conclusion about the effect of birch pollen AIT on FA-QoL. Taken together, no firm conclusions can be drawn, and future research is warranted that uses robust clinical studies that take into account the abovementioned aspects, including the long-term effects.

## References

[1] Lake IR Jones NR Agnew M Goodess CM Giorgi F Hamaoui-Laguel L Climate change and future pollen allergy in Europe. Environ Health Perspect. (2017) 125(3):385. 10.1289/EHP173 27557093 PMC5332176

[2] Biedermann T Winther L Till SJ Panzner P Knulst A Valovirta E. Birch pollen allergy in Europe. Allergy. (2019) 74(7):1237–48. 10.1111/all.13758 30829410

[3] Matricardi PM Kleine-Tebbe J Hoffmann HJ Valenta R Hilger C Hofmaier S EAACI Molecular allergology user’s guide. Pediatr Allergy Immunol. (2016) 27(Suppl 23):1–250. 10.1111/pai.12563 27288833

[4] Werfel T Asero R Ballmer-Weber BK Beyer K Enrique E Knulst AC Position paper of the EAACI: food allergy due to immunological cross-reactions with common inhalant allergens. Allergy. (2015) 70(9):1079–90. 10.1111/all.12666 26095197

[5] Kleine-Tebbe J Wangorsch A Vogel L Crowell DN Haustein UF Vieths S. Severe oral allergy syndrome and anaphylactic reactions caused by a bet v 1-related PR-10 protein in soybean, SAM22. J Allergy Clin Immunol. (2002) 110(5):797–804. 10.1067/mai.2002.128946 12417891

[6] Skypala IJ Hunter H Krishna MT Rey-Garcia H Till SJ du Toit G BSACI guideline for the diagnosis and management of pollen food syndrome in the UK. Clin Exp Allergy. (2022) 52(9):1018–34. 10.1111/cea.14208 35975576

[7] Clayton J Skypala I. Late breaking poster session LB TPS 10–18. Allergy. (2016) 71:592–633. 10.1111/all.12979

[8] Boonpiyathad T Lao-Araya M Chiewchalermsri C Sangkanjanavanich S Morita H. Allergic rhinitis: what do we know about allergen-specific immunotherapy? Front Allergy. (2021) 2:1–22. 10.3389/falgy.2021.747323 PMC8974870 35387059

[9] Pavón-Romero GF Parra-Vargas MI Ramírez-Jiménez F Melgoza-Ruiz E Serrano-Pérez NH Teran LM. Allergen immunotherapy: current and future trends. Cells. (2022) 11:1–22. doi: 10.3390/cells11020212 10.3390/cells11020212 PMC8774202 35053328

[10] Sterne JAC Savović J Page MJ Elbers RG Blencowe NS Boutron I Rob 2: a revised tool for assessing risk of bias in randomised trials. Br Med J. (2019) 366(I4898):1–8. doi: 10.1136/bmj.l4898 10.1136/bmj.l4898 31462531

[11] Treudler R Franke A Schmiedeknecht A Ballmer-Weber B Worm M Werfel T BASALIT trial: double-blind placebo-controlled allergen immunotherapy with rBet v 1-FV in birch-related soya allergy. Allergy. (2017) 72(8):1243–53. 10.1111/all.13112 27998002

[12] Van Hoffen E Peeters KABM Van Neerven RJJ Van Der Tas CWH Zuidmeer L Van Ieperen-Van Dijk AG Effect of birch pollen-specific immunotherapy on birch pollen-related hazelnut allergy. J Allergy Clin Immunol. (2011) 127(1):100–1.e3. 10.1016/j.jaci.2010.08.021 20933256

[13] Bolhaar STHP Tiemessen MM Zuidmeer L Van Leeuwen A Hoffmann-Sommergruber K Bruijnzeel-Koomen CAFM Efficacy of birch-pollen immunotherapy on cross-reactive food allergy confirmed by skin tests and double-blind food challenges. Clin Exp Allergy. (2004) 34(5):761–9. 10.1111/j.1365-2222.2004.1939.x 15144469

[14] Hansen KS Khinchi MS Skov PS Bindslev-Jensen C Poulsen LK Malling HJ. Food allergy to apple and specific immunotherapy with birch pollen. Mol Nutr Food Res. (2004) 48(6):441–8. 10.1002/mnfr.200400037 15508179

[15] Till SJ Stage BS Skypala I Biedermann T. Potential treatment effect of the SQ tree SLIT-tablet on pollen food syndrome caused by apple. Allergy. (2020) 75:2059–61. 10.1111/all.14242 32086823

[16] Asero R. Effects of birch pollen-specific immunotherapy on apple allergy in birch pollen-hypersensitive patients. Clin Exp Allergy. (1998) 28(11):1368–73. 10.1046/j.1365-2222.1998.00399.x 9824409

[17] Bucher X Fichier WJ Dahinden CA Helbling A. Effect of tree pollen specific, subcutaneous immunotherapy on the oral allergy syndrome to apple and hazelnut. Allergy. (2004) 59(12):1272–6. 10.1111/j.1398-9995.2004.00626.x 15507095

[18] van der Valk J Nagl B van Wljk RG Bohle B de Jong N. The effect of birch pollen immunotherapy on apple and rmal d 1 challenges in adults with apple allergy. Nutrients. (2020) 12(2):1–11. 10.3390/nu12020519 PMC7071292 32085633

[19] Bergmann KC Wolf H Schnitker J. Effect of pollen-specific sublingual immunotherapy on oral allergy syndrome: an observational study. World Allergy Organ J. (2008) 1(5):79–84. 10.1097/WOX.0b013e3181752d1c 23282323 PMC3650975

[20] Kinaciyan T Jahn-Schmid B Radakovics A Zwölfer B Schreiber C Francis JN Successful sublingual immunotherapy with birch pollen has limited effects on concomitant food allergy to apple and the immune response to the bet v 1 homolog mal d 1. J Allergy Clin Immunol. (2007) 119(4):937–43. 10.1016/j.jaci.2006.11.010 17204315

[21] Dodig S Čepelak I. The potential of component-resolved diagnosis in laboratory diagnostics of allergy. Biochem Med (Zagreb). (2018) 28:1–9. 10.11613/BM.2018.020501 PMC5898957 29666553

[22] Roberts G Pfaar O Akdis CA Ansotegui IJ Durham SR Gerth van Wijk R EAACI guidelines on allergen immunotherapy: allergic rhinoconjunctivitis. Allergy. (2018) 73(4):765–98. 10.1111/all.13317 28940458

[23] Penagos M Eifan AO Durham SR Scadding GW. Duration of allergen immunotherapy for long-term efficacy in allergic rhinoconjunctivitis. Curr Treat Options Allergy. (2018) 5(3):275–90. 10.1007/s40521-018-0176-2 30221122 PMC6132438

[24] McGee RG. How to include patient-reported outcome measures in clinical trials. Curr Osteoporos Rep. (2020) 18:480–5. 10.1007/s11914-020-00611-5 32757118

[25] Sim K Mijakoski D Stoleski S del Rio PR Sammut P Le TM Outcomes for clinical trials of food allergy treatments. Ann Allergy Asthma Immunol. (2020) 125:35–42. doi: 10.1016/j.anai.2020.06.023 10.1016/j.anai.2020.06.023 32569834

[26] Lloyd M Dunn Galvin A Tang MLK. Measuring the impact of food immunotherapy on health-related quality of life in clinical trials. Front Allergy. (2022) 3:1–7. 10.3389/falgy.2022.941020 PMC9326481 35910858

[27] Lyons SA Burney PGJ Ballmer-Weber BK Fernandez-Rivas M Barreales L Clausen M Food allergy in adults: substantial variation in prevalence and causative foods across Europe. J Allergy Clin Immunol Pract. (2019) 7(6):1920–28.e11. 10.1016/j.jaip.2019.02.044 30898689

[28] Hoffmann-Sommergruber K Hilger C Santos A De Las Vecillas L Dramburg S. Molecular Allergology User’s Guide 2.0. Zurich, Switzerland: European Academy of Allergy and Clinical Immunology (2022).

[29] Asero R. How long does the effect of birch pollen injection SIT on apple allergy last? Allergy. (2003) 58(5):435–8. 10.1034/j.1398-9995.2003.00139.x 12752332

[30] Kinaciyan T Nagl B Faustmann S Frommlet F Kopp S Wolkersdorfer M Efficacy and safety of 4 months of sublingual immunotherapy with recombinant mal d 1 and bet v 1 in patients with birch pollen–related apple allergy. J Allergy Clin Immunol. (2018) 141(3):1002–8. 10.1016/j.jaci.2017.07.036 28870463

[31] Kopac P Rudin M Gentinetta T Gerber R Pichler C Hausmann O Continuous apple consumption induces oral tolerance in birch-pollen- associated apple allergy. Allergy. (2012) 67(2):280–5. 10.1111/j.1398-9995.2011.02744.x 22070352

[32] Inuo C Kondo Y Tanaka K Nakajima Y Nomura T Ando H Japanese cedar pollen-based subcutaneous immunotherapy decreases tomato fruit-specific basophil activation. Int Arch Allergy Immunol. (2015) 167(2):137–45. 10.1159/000437325 26302651

[33] Furci F Ricciardi L. Plant food allergy improvement after grass pollen sublingual immunotherapy: a case series. Pathogens. (2021) 10(11):1–6. 10.3390/pathogens10111412 PMC8618412 34832568

[34] Jaeschke R Singer J Guyatt GH. Measurement of health Status ascertaining the minimal clinically important difference. Control Clin Trials. (1989) 10:407–15. 10.1016/0197-2456(89)90005-6 2691207

