# Supplementary material for: The effect of subcutaneous and sublingual birch pollen immunotherapy on birch pollen–related food allergy: a systematic review
Source: Front Allergy. 2024 Jun 6;5:1360073. doi: 10.3389/falgy.2024.1360073 (PMC11187334; doi:10.3389/falgy.2024.1360073)
Supplement: Supplementary file 1 [file Datasheet1.docx]

**SUPPLEMENTAL**

**Supplemental S1a. Search strategy PubMed**

Search strategy for PubMed search performed on November 3^rd^ 2022.

Patient population: Birch pollen-related food allergy

1. food*[Title/Abstract] OR fruit[MeSH Terms] OR fruit*[Title/Abstract] OR rosaceae[MeSH Terms] OR rosaceae*[Title/Abstract] OR apple*[Title/Abstract] OR malus*[Title/Abstract] OR apricot*[Title/Abstract] OR cherr*[Title/Abstract] OR peach*[Title/Abstract] OR plum*[Title/Abstract] OR nectarin*[Title/Abstract] OR prunus*[Title/Abstract] OR pear*[Title/Abstract] OR pyrus*[Title/Abstract] OR actinidia[MeSH Terms] OR actinidia*[Title/Abstract] OR kiwi*[Title/Abstract] OR mangifera[MeSH Terms] OR mangifera*[Title/Abstract] OR mango*[Title/Abstract] OR diospyros[MeSH Terms] OR diospyros*[Title/Abstract] OR artocarpus[MeSH Terms] OR artocarpus*[Title/Abstract] OR jackfruit*[Title/Abstract] OR litchi[MeSH Terms] OR lychee*[Title/Abstract] OR litch*[Title/Abstract] OR leechee*[Title/Abstract] OR vitis[MeSH Terms] OR vitis*[Title/Abstract] OR grape*[Title/Abstract] OR ficus[MeSH Terms] OR ficus*[Title/Abstract] OR fig*[Title/Abstract] OR fabaceae[MeSH Terms] OR fabaceae*[Title/Abstract] OR legume*[Title/Abstract] OR soy food[MeSH Terms] OR soybeans[MeSH Terms] OR soy*[Title/Abstract] OR soj*[Title/Abstract] OR bean*[Title/Abstract] OR vegetable[MeSH Terms] OR vegetable*[Title/Abstract] OR daucus carota[MeSH Terms] OR daucus carota*[Title/Abstract] OR carrot*[Title/Abstract] OR apium graveolens[MeSH Terms] OR apium graveolen*[Title/Abstract] OR celer*[Title/Abstract] OR nuts[MeSH Terms] OR nut[Title/Abstract] OR nuts[Title/Abstract] OR corylus[MeSH Terms] OR corylus*[Title/Abstract] OR hazelnut*[Title/Abstract] OR arachis[MeSH Terms] OR arachis*[Title/Abstract] OR peanut*[Title/Abstract] OR solanum tuberosum[MeSH Terms] OR solanum tuberosum*[Title/Abstract] OR spices[MeSH Terms] OR spice*[Title/Abstract] OR herb*[Title/Abstract] OR sunflower seed*[Title/Abstract]

2. hypersensitivities[MeSH Terms] OR hypersensitiv*[Title/Abstract] OR allergens[MeSH Terms] OR allerg*[Title/Abstract] OR cross reactions[MeSH Terms] OR cross react*[Title/Abstract] OR crossreact*[Title/Abstract] OR ige mediat*[Title/Abstract] OR sensitis*[Title/Abstract] OR sensitis*[Title/Abstract]

3. 1 AND 2

4. food hypersensitivities[MeSH Terms]

5. 3 OR 4

6. pollen[MeSH Terms] OR pollen*[Title/Abstract] OR trees[MeSH Terms] OR tree*[Title/Abstract] OR orchard*[Title/Abstract] OR plane*[Title/Abstract] OR betulaceae[MeSH Terms] OR alnus*[Title/Abstract] OR alder*[Title/Abstract] OR betula*[Title/Abstract] OR birch*[Title/Abstract] OR corylus*[Title/Abstract] OR hazel*[Title/Abstract] OR filbert*[Title/Abstract] OR hornbeam*[Title/Abstract] OR quercus[MeSH Terms] OR quercus*[Title/Abstract] OR oak*[Title/Abstract]

7. 5 AND 6

8. oral allergy syndrom*[Title/Abstract] OR pollen food syndrom*[Title/Abstract] OR pollen-food syndrome*[Title/Abstract] OR pollen-related food*[ Title/Abstract] OR pollen related food*[Title/Abstract]

9. 7 OR 8

Intervention: Subcutaneous and/or sublingual immunotherapy

10. immunotherapy[MeSH Terms] OR immunotherap*[Title/Abstract] OR oral[Title/Abstract] OR injection*[MeSH Terms] OR subcutaneous*[Title/Abstract] OR sublingual immunotherapy[MeSH Terms] OR administration sublingual[MeSH Terms] OR sublingual*[Title/Abstract] OR SCIT[Title/Abstract] OR SLIT[Title/Abstract]

11. 6 AND 10

12. 9 AND 11

**Supplemental S1b. Search strategy Embase**

Search strategy for Embase search performed on November 3rd 2022.

Patient population: Birch pollen-related food allergy

1. food*:ab,ti OR 'fruit' OR fruit*:ab,ti OR 'rosaceae' OR rosaceae*:ab,ti OR 'apple' OR 'malus' OR 'apricot' OR 'cherry' OR 'peach' OR 'plum' OR 'nectarine' OR 'prunus' OR 'pear' OR 'pyrus' OR 'actinidia' OR actinidia:ab,ti OR 'kiwifruit' OR kiwi:ab,ti OR 'mangifera' OR mangifera:ab,ti OR 'mango' OR 'diospyros' OR diospyros*:ab,ti OR 'artocarpus' OR artocarpus:ab,ti OR jackfruit:ab,ti OR 'lychee' OR litchi:ti,ab OR litch*:ti,ab OR leechee*:ab,ti OR 'vitis' OR vitis*:ab,ti OR 'grape' OR grape:ab,ti OR 'ficus' OR ficus*:ab,ti OR fig*:ab,ti OR 'fabaceae' OR fabaceae:ab,ti OR 'legume' OR legume*:ab,ti OR 'soy food' OR 'soybean' OR soy*:ti,ab OR soj*:ab,ti OR 'bean' OR 'vegetable' OR vegetable:ab,ti OR 'carrot' OR carrot*:ab,ti OR 'celery' OR celery:ab,ti OR 'nut' OR nut*:ab,ti OR 'hazelnut' OR corylus*:ab,ti OR 'arachis' OR arachis*:ab,ti OR 'peanut' OR peanut:ab,ti OR 'potato' OR tuberosum*:ab,ti OR 'spice' OR spice*:ab,ti OR 'herb' OR sunflowerseed*:ab,ti
2. 'hypersensitivity' OR hypersensitiv*:ab,ti OR 'allergen' OR allerg*:ab,ti OR 'cross reaction' OR crossreact*:ab,ti OR 'immunoglobulin E' OR 'immediate type hypersensitivity' OR 'sensitization' OR sensitis*:ti,ab
3. 1 AND 2
4. 'food allergy'
5. 3 OR 4
6. 'pollen' OR pollen:ab,ti OR 'tree' OR tree*:ab,ti OR 'orchard' OR plane:ab,ti OR 'betulaceae' OR alnus*:ab,ti OR 'alder' OR betula*:ab,ti OR 'birch' OR 'hazelnut' OR corylus*:ab,ti OR hazel*:ab,ti OR filbert*:ab,ti OR hornbeam*:ab,ti OR 'oak' OR quercus*:ab,ti
7. 5 AND 6
8. 'oral allergy syndrome' OR 'pollen food syndrome' OR 'pollen-food syndrome*':ab,ti OR 'pollen-related food*':ab,ti OR 'pollen related food*':ab,ti
9. 7 OR 8

Intervention: Subcutaneous and/or sublingual immunotherapy

1. 'immunotherapy' OR immunotherap*:ab,ti OR oral*:ab,ti OR 'injection' OR subcutaneous*:ab,ti OR 'subcutaneous drug administration' OR 'sublingual immunotherapy' OR 'sublingual drug administration' OR sublingual*:ab,ti OR 'subcutaneous immunotherapy' OR scit*:ab,ti OR slit*:ab,ti
2. 6 AND 10
3. 9 AND 11

**Supplemental S1c. Search strategy Cochrane**

Search strategy for Cochrane search performed on November 3rd 2022.

Patient population: Birch pollen-related food allergy

1. [mh ‘soy foods’] or [mh dauces carota] or [solanum tuberosum] or [mh fruit] or [mh artocarpus] or [mh rosacea] or [mh litchi] or [mh actinidia] or [mh vitis] or [mh mangifera] or [mh ficus] or [mh malus] or [mh fabaceae] or [mh diospyros] or [mh soybeans] or [mh vegetable] or [mh apium] or [mh nuts] or [mh corylus] or [mh arachis] or [mh spices] or (food* or fruit* or rosaceae* or apple* or malus* or apricot* or cherr* or peach* or plum* or nectarin* or prunus* or pear* or pyrus* or actinidia* or kiwi* or mangifera* or mango* or diospyros* or artocarpus* or jackfruit* or lychee* or litch* or leechee* or vitis* or grape* or ficus* or fig* or fabaceae* or legume* or soy* or soj* or bean* or vegetable* or ''daucus carota''* or carrot* or ''apium graveolen''* or celer* or nut or nuts or corylus* or hazelnut* or arachis* or peanut* or ''solanum tuberosum''* or spice* or herb* or ''sunflower seed''*):ti,ab,kw
2. [mh ‘cross react’] or [mh hypersensitivity] or [mh allergens] or (hypersensitiv* or ''cross react*'' or ''ige mediat*'' or allerg* or crossreact* or sensitis*):ti,ab,kw
3. 1 AND 2
4. [mh food hypersensitivity]
5. 3 OR 4
6. [mh pollen] or [mh trees] or [mh betulaceae] or [mh quercus] or (pollen* or tree* or orchard* or plane* or alnus* or alder* or betula* or birch* or corylus* or hazel* or filbert* or hornbeam* or quercus* or oak*):ti,ab,kw
7. 5 AND 6
8. ("oral allergy syndrome" or ''pollen food syndrome'' or ‘’pollen-food syndrome’’* or ‘’pollen-related food’’* or ‘’pollen related food’’):ti,ab,kw
9. 7 OR 8

Intervention: Subcutaneous and/or sublingual immunotherapy

1. 'immunotherapy' OR immunotherap*:ti,ab,kw OR oral*:ti,ab,kw OR 'injections' OR subcutaneous*:ti,ab,kw OR 'injections, subcutaneous' OR 'sublingual immunotherapy' OR 'administration, sublingual' OR sublingual*:ti,ab,kw OR scit*:ti,ab,kw OR slit*:ti,ab,kw
2. 6 AND 10
3. 9 AND 11
